# Circulating Cell-Free DNA Reflects the Clonal Evolution of Breast Cancer Tumors

**DOI:** 10.3390/cancers14051332

**Published:** 2022-03-04

**Authors:** Jouni Kujala, Jaana M. Hartikainen, Maria Tengström, Reijo Sironen, Päivi Auvinen, Veli-Matti Kosma, Arto Mannermaa

**Affiliations:** 1 Institute of Clinical Medicine, Clinical Pathology and Forensic Medicine, University of Eastern Finland, FI-70211 Kuopio, Finland; jouni.kujala@uef.fi (J.K.); jaana.hartikainen@uef.fi (J.M.H.); reijo.sironen@kuh.fi (R.S.); veli-matti.kosma@uef.fi (V.-M.K.); 2 Multidisciplinary Cancer Research Area, University of Eastern Finland, FI-70211 Kuopio, Finland; 3 Cancer Center, Department of Oncology, Kuopio University Hospital, FI-70029 Kuopio, Finland; maria.tengstrom@kuh.fi (M.T.); paivi.auvinen@kuh.fi (P.A.); 4 Department of Clinical Pathology, Kuopio University Hospital, FI-70029 Kuopio, Finland; 5 Biobank of Eastern Finland, Kuopio University Hospital, FI-70029 Kuopio, Finland

**Keywords:** liquid biopsy, tumor evolution, intratumoral heterogeneity, sequencing, biomarker, recurrence, metastasis

## Abstract

**Simple Summary:**

Liquid biopsy of cell-free DNA (cfDNA) is proposed as potential method for the early detection of breast cancer (BC) metastases and following the clonal evolution of BC. Though the use of liquid biopsy is widely discussed, only few studies have demonstrated such usage so far. The aim of this study was to evaluate how accurately cfDNA resembles the genetic profile of tumor DNA and how liquid biopsy reflects the clonal evolution of 18 Eastern-Finnish BC cases with locoregional or distant metastases. Although notable discordance between the sequenced cfDNA and tumor DNA was observed, our results show that liquid biopsy reflects the heterogeneity and clonal evolution of BC and may help to identify potential driver variants and therapeutic targets that are not detected with the sequencing of tumor DNA. This information may be used to detect metastatic BC earlier and to support decision-making in clinical practice.

**Abstract:**

Liquid biopsy of cell-free DNA (cfDNA) is proposed as a potential method for the early detection of breast cancer (BC) metastases and following the clonal evolution of BC. Though the use of liquid biopsy is a widely discussed topic in the field, only a few studies have demonstrated such usage so far. We sequenced the DNA of matched primary tumor and metastatic sites together with the matched cfDNA samples from 18 Eastern Finnish BC patients and investigated how well cfDNA reflected the clonal evolution of BC interpreted from tumor DNA. On average, liquid biopsy detected 56.2 ± 7.2% of the somatic variants that were present either in the matched primary tumor or metastatic sites. Despite the high discordance observed between matched samples, liquid biopsy was found to reflect the clonal evolution of BC and identify novel driver variants and therapeutic targets absent from the tumor DNA. Tumor-specific somatic variants were detected in cfDNA at the time of diagnosis and 8.4 ± 2.4 months prior to detection of locoregional recurrence or distant metastases. Our results demonstrate that the sequencing of cfDNA may be used for the early detection of locoregional and distant BC metastases. Observed discordance between tumor DNA sequencing and liquid biopsy supports the parallel sequencing of cfDNA and tumor DNA to yield the most comprehensive overview for the genetic landscape of BC.

## 1. Introduction

Breast cancer (BC) is the most common cancer among women worldwide with over two million new cases diagnosed in 2020 [1]. Even though advances in diagnostics and treatment have improved the survival of BC patients, approximately 20% of patients will eventually develop recurrence or metastatic disease [2,3].

Risk for recurrence can be estimated with clinical and molecular characteristics of primary tumor and wide range of scoring methods have been developed [4] to utilize this information and evaluate the response and need for adjuvant therapies which are given to destroy the microscopic dissemination of cancer cells. Adjuvant therapies are given with curative intention, and they have significantly improved the outcome of BC patients [5]. Locoregional recurrences (LRs) can be curatively treated, but it is not known at which step systemic spread of cancer cells is beyond curative treatment and if it is possible to detect the systemic recurrence when BC would still be potentially curable.

One of the main causes that complicates the prediction of clinical outcome of BC is intratumoral heterogeneity (ITH) which refers to the presence of genetically distinct cancer cell populations within individual tumors. ITH results from clonal evolution of cancer cells where subclones with selective advantage providing somatic mutations become more common in tumor [6]. It is largely recognized that this variability is not always perfectly represented by tumor biopsies and some subclones may remain undetected. Since the used treatment is usually selected based on detected biomarkers, undetected subclones may survive and later manifest as recurrent or distant disease [7,8]. Moreover, the clonal structure of tumors tends to change over time which highlights the need for serial monitoring of heterogeneity to evaluate disease progression. This need cannot be filled by tumor biopsies due to their invasive nature.

Circulating cell-free DNA (cfDNA) refers to fragmented DNA that is released into circulation by cell-death or active secretion [9]. Small fractions of cfDNA are shown to originate from tumor cells and carry tumor-specific somatic variants, thus enabling minimally invasive, rapid, and easily repeated methods to identify clinically relevant somatic variants from blood samples [10]. Often referred as liquid biopsy, the analysis of cfDNA is proposed as an alternative method to genotype BC tumors and overcome problems related to ITH and early detection of BC recurrence and metastases [11,12]. Although the potential usage of liquid biopsy for following the clonal evolution of BC has been widely discussed during the past few years, only few studies [13,14,15,16] have demonstrated such usage so far.

Here, we sequenced the DNA of matched primary tumor and metastatic sites together with the matched cfDNA to investigate how accurately liquid biopsy reflects the ITH and clonal evolution of Eastern-Finnish BC cases with LR or distant metastases. Our results provide further evidence to support the use of liquid biopsy to estimate ITH and progression of clonal evolution.

## 2. Materials and Methods

### 2.1. Patients, Sample Material and Clinical Data

Clinical data, tumor, blood, and serum samples were obtained from the Kuopio Breast Cancer Project (KBCP) [17,18,19] and Itä-Länsi Rintasyöpäprojekti (ILRS), prospective population-based BC studies conducted in 1990–1995 and 2010–2014 in Eastern Finland. The research projects were advocated by the Research Ethics Committee of the University of Eastern Finland (UEF) and Kuopio University Hospital. All study participants signed written, informed consent to participate. For this study, we selected a total of 18 BC cases from the KBCP and ILRS cohorts based on available sample material and clinical picture of the disease.

We selected nine KBCP cases with a stage N0 disease who had developed LR recurrence or distant metastasis despite the good initial prognosis. Median recurrence-free survival (RFS) within selected cases was 3.6 years. Clinicopathological characteristics of patients are presented in Table 1. Blood, serum, and either formalin-fixed paraffin-embedded (FFPE) sections or fresh frozen (FF) tissues from the primary tumor and metastatic sites were available from all cases. Serum samples were collected at the time of diagnosis and at the latest follow-up on average of 8.4 ± 2.4 months prior to the detection of recurrence.

Nine selected ILRS cases were diagnosed with a stage N1-N3 disease. Either FFPE or FF samples from primary tumor and metastatic sites were available from all cases. By the time of the most recent follow-up, four ILRS cases had developed either LR recurrence or distant metastasis with a median RFS of 6.0 months. Unlike KBCP, ILRS did not contain follow-up serum samples or samples from LR recurrence or distant metastases. Analysis of ILRS cases was therefore focused on samples collected at the time of diagnosis.

### 2.2. DNA Isolation

QIAamp Circulating Nucleic Acid kit (Qiagen, Hilden, Germany) was used according to manufacturer’s protocol to isolate cfDNA from patient serum samples. Genomic DNA from tumor formalin-fixed paraffin-embedded (FFPE) sections, fresh frozen samples (FF) and blood samples were isolated with a High Pure FFPET DNA Isolation Kit (Roche Diagnostics, Indianapolis, IN, USA) and QIAamp DNA Blood Midi/Mini Kit (Qiagen) according to manufacturer’s protocol. Quality and concentration of DNA was assessed with Nanodrop ND-1000 (Thermo Fischer Scientific, Waltham, MA, USA) and Qubit 2.0 with a Qubit dsDNA BR Assay Kit (Invitrogen, Carlsbad, CA, USA). Quality and fragmentation of cfDNA was further analyzed with the TapeStation 4200 electrophoresis system with a D5000 High Sensitivity ScreenTape Assay (Agilent Technologies, Santa Clara, CA, USA) to identify possible gDNA contaminations (Figure S1).

### 2.3. Library Preparation and Sequencing

Sequencing libraries from cfDNA samples were prepared using the QIAseq cfDNA Library Kit (Qiagen) according to the manufacturer’s protocol. Libraries with unique molecular indices were pooled with xGen Universal blocking oligos (Integrated DNA Technologies, Coralville, IA, USA) and custom SureSelectXT2 target capture baits (Agilent Technologies) targeting 105 genes associated with metastatic BC (Table S1). The hybrid capture reaction was performed with the SureSelectXT2 Target Enrichment System Kit (Agilent Technologies) according to manufacturer’s protocol. Captured cfDNA libraries were on-bead amplified with GeneRead DNA I Amp Kit (Qiagen) according to manufacturer’s protocol and further purified with Agencourt AMPure XP beads (Beckman Coulter, Brea, CA, USA).

Sequencing libraries from FFPE, FF and blood-derived gDNA were prepared and enriched using the HaloPlexHS Target Enrichment System (Agilent Technologies) with custom amplicons targeting 39 genes (Table S2) according to manufacturer’s protocol. Agilent NGS FFPE QC Kit (Cat No. G9700B, Agilent Technologies) was used according to the manufacturer’s protocol to determine the DNA integrity scores and the amount of input FFPE-derived gDNA for the library preparation.

Molarity of libraries was quantified with the Bioanalyzer High sensitivity DNA Kit (Agilent Technologies) and equimolarly pooled libraries were sequenced with Illumina NextSeq or MiSeq sequencing platforms (Illumina, San Diego, CA, USA) located at the Genome Center of Eastern Finland, UEF, Kuopio, Finland.

### 2.4. Bioinformatics and Variant Calling

Paired-end reads were trimmed with cutadapt [20] and mapped to the hg19 reference genome with BWA-MEM [21]. Mapped reads with a Phred quality score <20 were excluded and remaining reads were sorted and indexed with SAMtools [22]. Local realignment was performed with GATK IndelRealigner [23] tools to minimize the number of mismatching bases across all reads. Read quality was assessed with FastQC [24] and Picard CollectHsMetrics [25].

Variant calling for somatic single nucleotide variants (SNVs) and indels was performed using VarScan2 [26]; the minimum allelic frequency for somatic mutations was set to 0.01 and at least 10 variant-supporting reads were required to retain the variant. Variants reported in Finnish population in the ExAC project [27] and matched blood samples were filtered to exclude germline variants. Called variants were annotated with ANNOVAR [28] and Cancer Genome Interpreter (CGI) [29]. In the case of CGI, database searches were focused on BC-specific results. Copy-number variations were identified with CNVkit [30]. The computational analyses were run on the servers provided by the Bioinformatics Center, UEF, Kuopio, Finland.

### 2.5. Tumor Evolution Modeling

Clonal evolution of the sequenced tumors was estimated with PhyloWGS v.1.0-rc1 [31] and SciClone v.1.1.0 [32] with default parameters. Manually curated variant and CNV data from the sequenced tumors were used as an input and results from both tools were interpreted by the authors. In the case of PhyloWGS, a tree model with the lowest normalized log likelihood (nLgLH) score was selected as the best candidate for evolution model to which other tree models were compared. The reported tree models represent the interpreted consensus of generated tree models.

### 2.6. Statistical Analysis

Linear correlation between two variables was estimated with the Pearson correlation coefficient. A *p*-value of 0.05 (two-sided) or less was considered statistically significant. Numerical values are presented as the mean ± standard error of the mean (SEM). Differences in group means were compared with paired or unpaired t-tests with Bonferroni correction for multiple testing when applicable. Statistical analyses were focused on genes included both in the HaloPlexHS and SureSelectXT2 panels. Concordance between tumor and cfDNA was calculated by counting the number of somatic variants that were detected both in the tumor and cfDNA and dividing it by the somatic variant count of the matched tumor.

## 3. Results

### 3.1. Sequencing Performance

Ratios of targeted bases covered with more than 100 reads after read processing were 92.5 ± 0.3%, 84.4 ± 0.8%, 82.1 ± 2.0% and 93.2 ± 0.8% for cfDNA, blood, and tumor FFPE and FF samples, respectively (Figure S2). Achieved mean target coverages after read processing were 345 ± 24X for blood samples, 615 ± 23X and 1125 ± 27X for tumor FFPE and FF samples, and 3890 ± 95X for cfDNA samples.

### 3.2. Detected Somatic Variants

**Figure 1:**
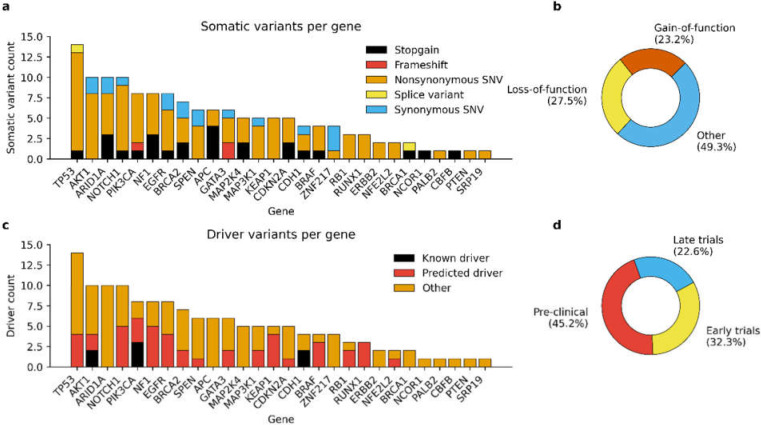
Functional and clinical relevance of detected somatic variants. (**a**) Distribution of somatic variants per gene is typical for BC and highlights the high mutation burden of genes *TP53*, *AKT1*, *ARID1A*, and *NOTCH1*. (**b**) Functional consequence of somatic variants as predicted by the CGI. Almost half of the variants were predicted to have gain-of-function or loss-of-function type consequences for the gene product. (**c**) CGI identified 56 known or predicted driver variants. In general, these variants were well prominent in the generated tumor evolution models, thus supporting the selective advantage provided by drivers. (**d**) Identified drug sensitivities as reported by the CGI. Most drugs that are reported to be sensitive for the detected somatic variants are still in pre-clinical or early clinical trials.

On average, we detected 3.6 ± 0.5 SNVs and indels (10.6 ± 1.5 variants/Mbp) per sequenced primary tumor and 4.5 ± 0.5 somatic variants (13.3 ± 1.8 variants/Mbp) per sequenced metastases. Corresponding variant frequencies for cfDNAs collected at the time of diagnosis and prior to the diagnosis of LR recurrence or distant metastases were 7.2 ± 1.4 variants (14.6 ± 3.0 variants/Mbp) and 4.6 ± 0.6 variants (10.6 ± 1.2 variants/Mbp). The number of somatic variants was generally higher in cases with higher tumor grade and lymph node status, although differences were not statistically significant. The most mutated genes across all sample groups and their share from overall somatic variant count were *TP53* (13.7%), *AKT1* (9.4%), *PIK3CA* (9.4%), *ARID1A* (7.9%), and *NOTCH1* (7.9%) (Figure 1a).

In total, the variant calling yielded 143 unique SNVs and indels in the sequenced samples (Figure 1a). Approximately half of the variants (50.7%) of the variants were predicted to have loss-of-function or gain-of-function type functional consequences (Figure 1b) and almost 40% (39.8%) of the variants were annotated either as known or potential driver mutations by CGI (Figure 1c). Detected variants were associated with reported drug sensitivity for 31 targeted therapies that are currently in pre-clinical or clinical trials for the treatment of BC (Figure 1d). Correspondingly, eight variants were reported to be associated with acquired drug resistance.

Our sequencing pipeline was able to detect the most prominent CNVs, such as the amplifications of *MYC* (22.2% of cases) and *HER2* (22.2%), from the tumor DNA sequencing data, although the used gene panel was not specifically designed to identify CNVs from sequenced samples. All *HER2* amplifications were detected in cases that were previously identified as HER2 positive cases with immunohistochemistry and chromogenic in situ hybridization (CISH). Corresponding CNVs were detectable in matched cfDNA as well but could not pass quality filtering due to high background noise. CNV results should be therefore considered tentative in the matched cfDNA.

### 3.3. Multi-Region Sequencing Demonstrates the Clonal Evolution of Metastatic BC

**Figure 2:**
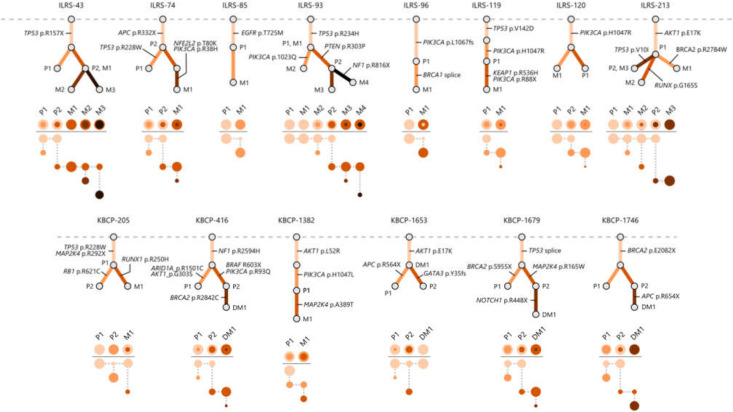
Evolutionary trees constructed from the multi-region sequencing results illustrate how locoregional metastases and distant metastases emerge from primary tumors and acquire new somatic variants over time. Each node in the trees represents a single subclone detected in the tumor samples, top node representing a hypothetical healthy cells where somatic variants are not detected. Abbreviations P, M, and DM with a running number refer to sequenced primary tumor, locoregional metastases, and distant metastases. The clonal structure of sequenced samples is visualized below the evolutionary trees as color-coded circles where the area of the circles is proportional to the estimated cancer cell fraction and each color represents a separate subclone. Predicted driver variants and variants with known pathogenicity are shown next to the evolutionary trees.

Matched primary tumors and metastatic sites shared at least one common somatic variant in 82.4% (14 out of 17) of the cases which was considered as a proof of the common ancestor and allowed us to construct the evolutionary trees for 14 BC cases (Figure 2). The evolutionary trees illustrate the accumulation of somatic variants over time and share the characteristics of both linear and branched tumor evolution. No metastasis-to-metastasis seeding was confirmed within the dataset. Evolutionary models underline the heterogenic nature of the BC as the clonal structure of the primary tumor and its metastatic sites varied considerably in few cases.

### 3.4. Liquid Biopsy Detects the Most Prominent Tumor-Specific Somatic Variants

**Figure 3:**
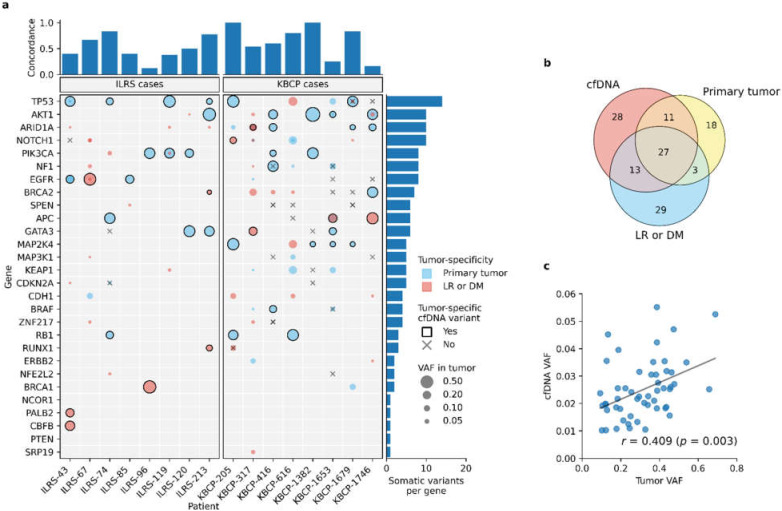
Concordance between tumor DNA and cfDNA sequencing results. (**a**) Comparison between matched tumor DNA and cfDNA samples visualized as a coMut plot. Each row represents one gene and each column represents one BC case. Bar plot at the top represent the observed concordance between matched samples while bar plot at the right represents the somatic variant count per gene. Circles within matrix cells represent somatic variants detected with the sequencing, blue and red circles corresponding to tumor-specific variants that originated either from the primary tumor or metastatic sites. Size of the circle corresponds to the VAF at the tumor. Circles with black line correspond to variants that were detected with the liquid biopsy as well while crosses represent variants that were detected only in the cfDNA. In general, liquid biopsy detects somatic variants that are represented with a relatively high VAF in primary tumor, locoregional recurrence (LR), or distant metastases (DM). (**b**) Venn diagram representation of detected somatic variant counts in different sample types illustrates how sequencing results of primary tumor, LRs and distant metastases, and cfDNA overlap with each other. (**c**) Statistically significant Pearson correlation (*p* = 0.003) was observed between matched tumor VAFs and cfDNA VAFs, thus supporting the idea that VAF in cfDNA reflects the clonal structure of primary tumor. All samples were included in the analysis.

Tumor-specific somatic variants were detected in 94.4% (17 out of 18) of cfDNA samples that were collected at the time of diagnosis (Figure 3a). As expected, detected variant allele frequencies (VAFs) were significantly lower (*p* < 0.001, unpaired *t*-test) in cfDNA than matched primary tumor samples and strong correlation was observed between primary tumor and cfDNA VAFs (*r* = 0.409, *p* = 0.003). The observed correlation was consistent with the observation that somatic variants that were presented in the tumor with higher VAFs were more commonly detected in the matched cfDNA samples as well.

On average, liquid biopsy detected 56.2 ± 7.2% of the somatic variants that were present either in the matched primary tumor or its metastatic sites, thus showing notable discordance between sequencing results. Observed discordance was mainly explained by the somatic variants that were presented with the low VAF (≤5%) in the primary tumor and metastatic sites but were not detected in the matched cfDNA. Likewise, an average of 17.0 ± 4.4% of the somatic variants detected in the cfDNA were absent in the matched primary tumor or its metastatic sites.

### 3.5. Serial Sequencing of cfDNA Reflects Changes in Clonal Evolution

**Figure 4:**
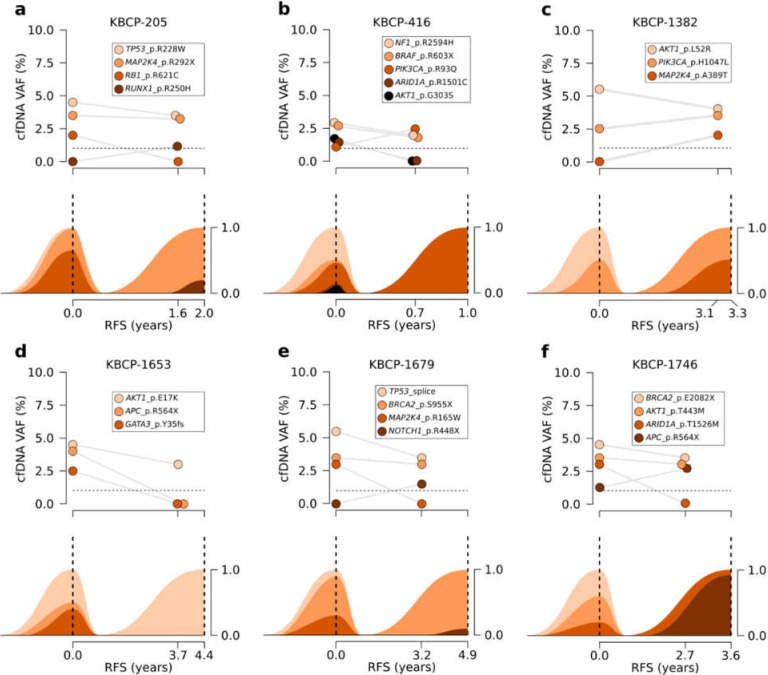
Clonal evolution of KBCP cases and corresponding VAFs in the serial cfDNA samples. Lineplots represent the detected VAF in sequenced cfDNA samples at the time of diagnosis and at the latest follow-up prior to the detection of LR or distant metastases. Fishplots below represent the corresponding clonal evolution in matched tumor samples. Plots (**a**–**f**) represent the disease progression of separate BC cases and illustrate how detected cfDNA VAFs follow the corresponding tumor VAFs especially in the case of trunk variants which makes it possible to detect bottlenecks during the disease progression with liquid biopsy. An interesting example is case KBCP-1746 (**f**) where the sequencing of cfDNA is inconsistent with the tumor evolution model and suggests that tumor samples do not represent ITH reliably.

We compared the presence of somatic variants in the serial cfDNA samples to interpreted clonal evolution of tumors to estimate how liquid biopsy follows the clonal evolution of BC. While the liquid biopsy’s ability to detect rare variants was limited, clonal changes of trunk variants, i.e., variants that occur at an early stage of tumor evolution and are present in all cancer cells, were well reflected by the cfDNA (Figure 4). These variants were often associated with known or predicted pathogenicity and driverness.

For example, the primary tumor of case KBCP-205 was characterized by its trunk driver mutation *TP53*_p.R228W and its two subclones carrying *RB1*_p.R621C and *MAP2K4*_p.R292X variants (Figure 4a). *RB1*-mutated subclone was the prominent subclone and accounted approximately 65.6% of all cancer cells while the *MAP2K4*-mutated subclone played a minor role. The clonal structure of primary tumor was supported by the cfDNA that carried indicated tumor-specific somatic variants at the time of diagnosis. The second cfDNA sample was collected at regular follow-up approximately 1.6 years after the primary diagnosis; sequencing detected tumor-specific variants *TP53*_p.R228W and *MAP2K4*_p.R292X but not variant *RB1*_p.R621C that was detected in the cfDNA at the time of diagnosis. Distant metastasis was diagnosed five months later and sequencing of metastatic site confirmed that the metastasis carried variants *TP53*_p.R228W and *MAP2K4*_p.R292X but not *RB1*_p.R621C, thus suggesting that the metastasis originated from the undetected subclone.

Another interesting example is case KBCP-1746 (Figure 4f) where primary tumor consisted of two major subclones that were both characterized by the trunk driver mutation *BRCA2*_p.E2082X. First subclone was further characterized by the passenger mutation *AKT1*_p.T443M while the second subclone was characterized by the passenger mutation *ARID1A*_p.T1526M. Distant metastasis was diagnosed 3.6 years after the primary diagnosis and sequencing of metastatic site suggested that the metastasis originated from the second subclone that had acquired a new driver mutation *APC*_p.R564X. Sequencing of cfDNA provided an alternative model for the disease progression; in addition to trunk variants *BRCA2*_p.E2082X, *AKT1*_p.T443M, and *ARID1A*_p.T1526M, cfDNA had also carried variant *APC*_p.R564X at the time of diagnosis. This suggests that variant was present already at the time of diagnosis but was not present in the sequenced primary tumor. Follow-up cfDNA collected approximately nine months before the detection of distant metastasis carried the same set of mutations with the exception of *ARID1A*_p.T1526M.

## 4. Discussion

Molecular characterization of BC strongly relies on primary tumors while metastatic sites often receive less attention. Considering the heterogenic nature of BC, further analysis of metastatic sites could be beneficial when assessing the clonal structure of disease and selecting the most effective treatments [33] but the invasive nature of sampling often makes it non-feasible to collect such samples. Our study demonstrates that liquid biopsy can, to some extent, help to overcome these constraints and help to provide more comprehensive insights into clonal structure of BC.

Similar to recent studies [34,35], our results show that tumor-specific somatic variants can be detected with the liquid biopsy at least few months before the LR recurrence or distant metastasis was detected at regular follow-up. It is tempting to think that the presence of tumor-specific variants in the follow-up cfDNA could act as an indication for more comprehensive examination and enable earlier diagnosis of the recurrent BC when it is still potentially curable. What remains open is the exact time window when the detection of tumor-specific variants is possible and when liquid biopsy should be ideally performed. Samples taken at the time of diagnosis and surgery are usually well available and feasible to analyze due to higher cfDNA concentrations and prognostic potential. From the follow-up perspective, post-operative and post-treatment samples might provide more information but they are often more challenging to analyze as cancer treatments are shown to induce the release of cfDNA from healthy tissues and decrease the concentration of tumor-originating cfDNA for a few weeks [36,37]. The ideal approach to performing liquid biopsy could be to use pre-operative cfDNA samples as baseline samples and collect follow-up samples with relatively short intervals especially during the first five years of follow-up when most LR recurrences and distant metastases of aggressive BC cases are observed.

Our results show that changes in the clonal evolution of BC can be tracked with serial sequencing of cfDNA. Liquid biopsy was able to detect the most prominent tumor-specific variants which were associated with predicted driverness and, in a few cases, with reported responsiveness or resistance for the targeted therapies that are currently developed for BC. For example, detected oncogenic *PIK3CA* variants were associated with reported responsiveness for PI3K/AKT/mTOR pathway inhibitors such as everolimus [38], while oncogenic *TP53* variants were associated with reported resistance for CDK4/6 inhibitor bicalutamide in a pre-clinical trial [39]. Our study did not allow the evaluation of treatment responses, but recent studies have successfully demonstrated the use of liquid biopsy to monitor treatment responses and developing chemoresistance [40]. Together, these results demonstrate the versatility and potential of liquid biopsy as it could be potentially used to monitor disease progression and treatment response, thus guiding clinicians in the decision-making process.

While the interpretation of tumor-specific variants is relatively straightforward, it is far more challenging to estimate the clinical significance of variants that cannot be directly linked to tumor. For example, we reported case KBCP-1746 where baseline cfDNA carried a truncating variant that was not detected in the primary tumor but was later detected in the distant metastasis, thus suggesting that the metastasis originated from the undetected subclone of primary tumor. Primary tumors are often used as a reference to which liquid biopsy is compared and our results remind that direct comparison with primary is not always unproblematic. Even though the functional and clinical relevance of variants can be estimated with continuously improving accuracy it is still difficult to estimate which subclone will eventually manifest itself as metastasis—especially if we cannot be sure that variant is originating from tumor cells. Serial sequencing of cfDNA could help us to identify variants that are gaining more of a foothold on tumor and metastatic sites and thus provide new dimension for the clinical evaluation of variants.

Although our results demonstrate the potential usage of liquid biopsy, we cannot ignore the varying levels of discordance between matched tumor and cfDNA samples. This discordance has been shown to arise both biological and preanalytical factors [41] and it has recently raised justified concerns about the accuracy of liquid biopsy [42]. Although we paid special attention to sample preprocessing, quality control, and variant calling to minimize the risk of gDNA contamination and effect of formalin-induced variants, we cannot completely exclude the effect of preanalytical factors in our study. Moreover, observed discordance will likely reflect the limitations of sequencing and variant calling. Liquid biopsy failed to detect tumor-specific variants that were presented in the tumor with low VAF, which strongly suggests that more sensitive sequencing approaches are needed to capture these variants reliably. In practice, this often means even deeper sequencing or the use of more sophisticated methods such as unique molecular identifiers [43]. At the same, there is an apparent pressure to perform liquid biopsy as cost-effectively as possible so that the liquid biopsy can truly compete with tumor DNA sequencing which is often more affordable option in clinics. At the moment, our results support tumor DNA sequencing and liquid biopsy as complementary methods, not as substitutes for each other and highlight the benefits of liquid biopsy to be the most apparent in situations where tumor sampling is not either feasible or possible.

## 5. Conclusions

Our results show that the tumor-specific somatic variants of BC tumor and its metastatic sites can be detected in the cfDNA both at the time of diagnosis and prior to locoregional recurrence or distant metastasis, thus providing further evidence to support the use of liquid biopsy in the early detection of BC recurrences and metastases. Results of cfDNA sequencing were consistent with the interpreted clonal evolution with an acceptable accuracy, thus demonstrating the use of liquid biopsy in the clonal evolution tracking. Taking account the high discordance in the spectrum of detected variants that was observed between matched tumors and cfDNA, our result show tumor DNA sequencing and liquid biopsy as complementary methods and support their parallel use to achieve the most comprehensive overview for the ITH and clonal evolution of BC.

## Figures and Tables

**Table 1:** Clinicopathological characteristics of patients.

Variable	Grouping	KBCP Cases	ILRS Cases	All Cases
Age at diagnosis	≤39 years	0 (0.0%)	1 (11.1%)	1 (5.6%)
	40–49 years	1 (11.1%)	1 (11.1%)	2 (11.1%)
	50–59 years	6 (66.7%)	0 (0.0%)	6 (33.3%)
	60–69 years	2 (22.2%)	4 (44.5%)	6 (33.3%)
	≥70 years	0 (0.0%)	3 (33.3%)	3 (16.7%)
ER status	Positive	7 (77.8%)	6 (66.7%)	13 (72.2%)
	Negative	2 (22.2%)	3 (33.3%)	5 (27.8%)
PR status	Positive	7 (77.8%)	7 (77.8%)	14 (77.8%)
	Negative	2 (22.2%)	2 (22.2%)	4 (22.2%)
HER2 status	Positive	1 (11.1%)	3 (33.3%)	4 (22.2%)
	Negative	8 (88.9%)	6 (66.7%)	14 (77.8%)
Tumor grade	I	3 (33.3%)	0 (0.0%)	3 (16.7%)
	II	3 (33.3%)	4 (44.4%)	7 (38.9%)
	III	3 (33.3%)	5 (55.6%)	8 (44.4%)
Tumor size	T1	6 (66.7%)	1 (11.1%)	7 (38.9%)
	T2	3 (33.3%)	7 (77.8%)	10 (55.5%)
	T3	0 (0.0%)	1 (11.1%)	1 (5.6%)
Lymph node status	N0	9 (100.0%)	0 (0.0%)	9 (50.0%)
	N1	0 (0.0%)	6 (66.7%)	6 (33.3%)
	N2	0 (0.0%)	1 (11.1%)	1 (5.6%)
	N3	0 (0.0%)	2 (22.2%)	2 (11.1%)
Distant metastases	M0	9 (100.0%)	0 (0.0%)	9 (50.0%)
	M1	0 (0.0%)	9 (100.0%)	9 (50.0%)
Histological subtype	Ductal carcinoma	8 (88.8%)	9 (100.0%)	17 (94.4%)
	Tubular carcinoma	1 (11.2%)	0 (0.0%)	1 (5.6%)
Outcome	Locoregional recurrence	2 (22.2%)	1 (11.1%)	3 (16.7%)
	Distant metastasis	7 (77.8%)	3 (33.3%)	10 (55.5%)
	Disease-free	0 (0.0%)	5 (55.6%)	5 (27.8%)

Used abbreviations: KBCP, Kuopio Breast Cancer Project; ILRS, Itä-Länsi Rintasyöpäprojekti; ER, estrogen receptor; PR, progesterone receptor; HER2, human epidermal growth factor receptor 2.

## Data Availability

The datasets used and analyzed during the study are available from the corresponding author on reasonable request.

## Supplementary Materials

The following supporting information can be downloaded at: https://www.mdpi.com/article/10.3390/cancers14051332/s1, Figure S1: Summary of cfDNA quality control metrics; Figure S2: Sequencing performance; Table S1: Content of SureSelectXT2 gene panel; Table S2: Content of HaloPlexHS gene panel.
