# Supplementary material for: Circulating Cell-Free DNA Reflects the Clonal Evolution of Breast Cancer Tumors
Source: Cancers (Basel). 2022 Mar 4;14(5):1332. doi: 10.3390/cancers14051332 (PMC8909912; doi:10.3390/cancers14051332)
Supplement: Supplementary file 1 [file cancers-14-01332-s001.zip › FigureS1.pdf]

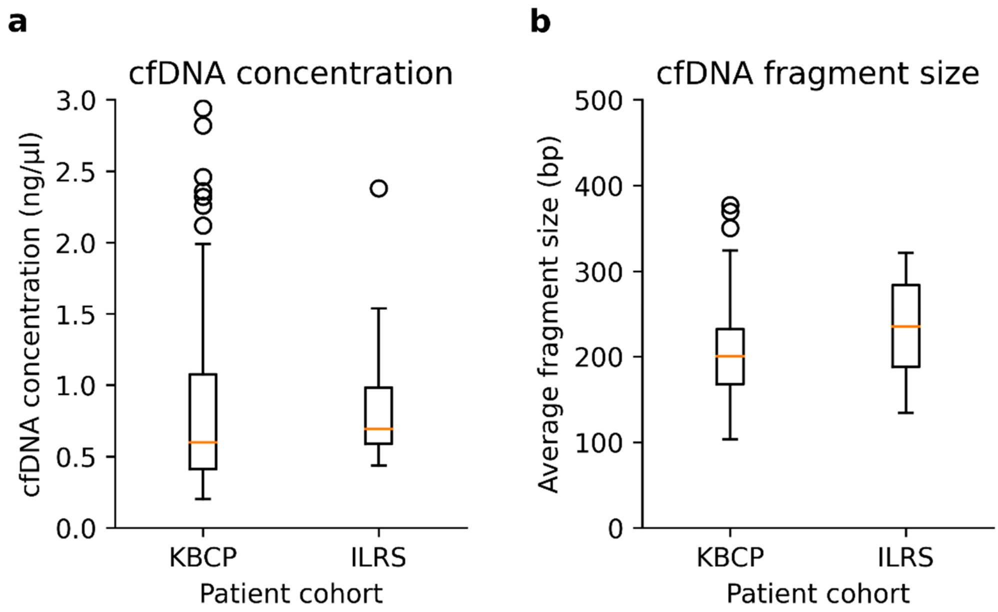

Supplementary Figure S1. Summary of cfDNA quality metrics measured with TapeStation 4200 automated electrophoresis system. Measured cfDNA concentration in KBCP and ILRS serum samples (a) and an average fragments size in isolated cfDNA samples (b). In general, quality of cfDNA samples was similar in both cohorts.
