# Supplementary material for: Circulating Cell-Free DNA Reflects the Clonal Evolution of Breast Cancer Tumors
Source: Cancers (Basel). 2022 Mar 4;14(5):1332. doi: 10.3390/cancers14051332 (PMC8909912; doi:10.3390/cancers14051332)
Supplement: Supplementary file 1 [file cancers-14-01332-s001.zip › FigureS2.pdf]

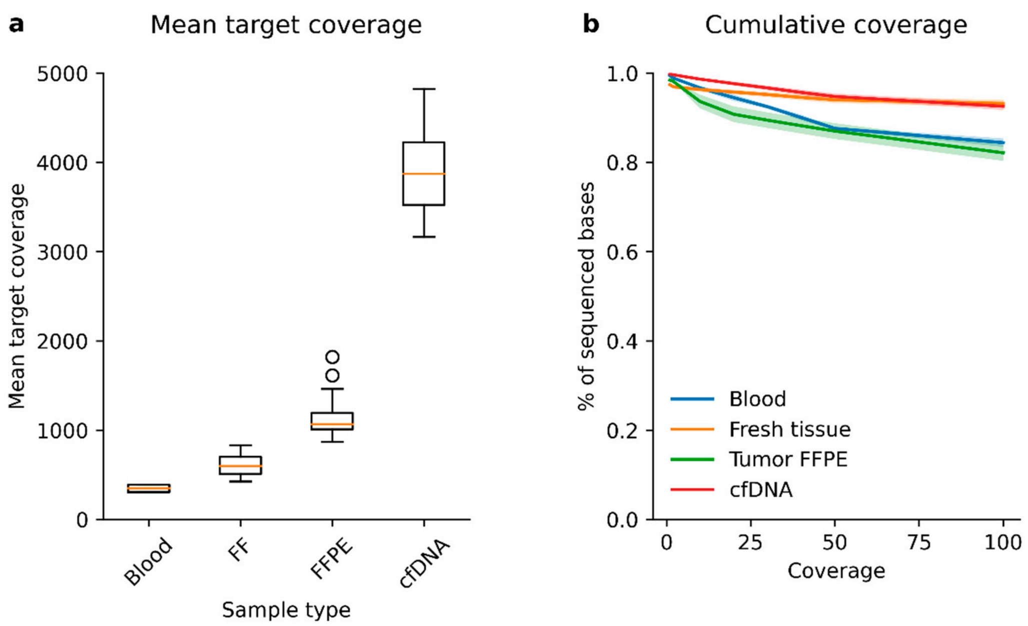

Supplementary Figure S2. Sequencing performance. Mean target coverage in different sample groups (a) and cumulative target base coverage on sequenced regions (b). Blood and FF samples were intentionally sequenced with lower sequencing depth than FFPE and cfDNA samples, in which the quality of sample material and detection of rare somatic variants required higher sequencing depth.
